# Intracellular proteome compartmentalization: a biotin ligase-based proximity labeling approach

**DOI:** 10.1186/s13578-021-00666-6

**Published:** 2021-08-23

**Authors:** Olanrewaju Ayodeji Durojaye

**Affiliations:** 1 School of Life Sciences, Department of Molecular and Cell Biology, University of Science and Technology of China, Hefei, China; 2 MOE Key Laboratory of Membraneless Organelle and Cellular Dynamics, Hefei National Laboratory for Physical Sciences at the Microscale, University of Science and Technology of China, Hefei, China; 3 Department of Chemical Sciences, Coal City University, Emene, Enugu Nigeria

**Keywords:** BioID Proximity labeling, Mass spectrometry, Proteome, APEX

## Abstract

Specialized biological processes occur in different regions and organelles of the cell. Additionally, the function of proteins correlate greatly with their interactions and subcellular localization. Understanding the mechanism underlying the specialized functions of cellular structures therefore requires a detailed identification of proteins within spatially defined domains of the cell. Furthermore, the identification of interacting proteins is also crucial for the elucidation of the underlying mechanism of complex cellular processes. Mass spectrometry methods have been utilized systematically for the characterization of the proteome of isolated organelles and protein interactors purified through affinity pull-down or following crosslinking. However, the available methods of purification have limited these approaches, as it is difficult to derive intact organelles of high purity in many circumstances. Furthermore, contamination that leads to the identification of false positive is widespread even when purification is possible. Here, we present a highlight of the BioID proximity labeling approach which has been used to effectively characterize the proteomic composition of several cellular compartments. In addition, an observed limitation of this method based on proteomic spatiotemporal dynamics, was also discussed.

**Dear editor**,

We have read with great interest the recent publication by Go et al. [1]; a study in which a proximity-dependent biotinylation approach was used in defining the proteomic composition of several compartments in living cells. Here, we report major highlights of the study and in addition discussed specific limitations of the approach based on the spatiotemporal dynamic nature of the human proteome.

The proximity labeling method has been developed to study the spatial compartmentalization of protein networks and their assembling pattern into functionally integrated complexes. This method involves the selective and covalent biotin tagging of neighbouring proteins in a living cell with the use of engineered enzymes. Isolation of the biotinylated proteins can then be carried out after cell lysis and mass spectrometry characterization. Proximity labeling has previously been applied in the mapping of different cell organelle components as well as in the identification of novel interactions with increased spatial specificity. These studies have shown that the proximity labeling approach is an efficient method for the dissection of molecular localization and interaction patterns with nanometer spatial resolution [2].

There are two major categories of the proximity labeling methods, both of which are linked to the type of enzyme used for catalysis. The peroxidase-based proximity labeling depends on the expression of an engineered HRP (horseradish peroxidase) or APEX (ascorbate peroxidase) in the tissues or cells of interest. HRP can alternatively be targeted to specific antigens on the cell surface through antibody conjugation. To start labeling, hydrogen peroxide is always added to tissues or cells for 1 min. The cells are then to be pre-loaded with biotin-phenol or its variants, such as desthiobiotin phenol, alkyne-phenol and BxxP. Biotin-phenol is oxidized by the peroxidase into phenoxyl radical which in turn reacts with neighboring proteins at their electron-rich side chains. Because the half-life of the phenoxyl radical is less than 1 ms, the intensity of labeling falls dramatically within few nanometers from the active site of the peroxidase. This generates a biotinylation contour map which can be analyzed by quantitative proteomics to produce a list of proteins that are ranked on the basis of proximity to the enzyme [3].

In the biotin ligase-based (BioID) proximity labeling approach, BirA* (a mutant biotin ligase from *E. coli*) is attached to a polypeptide of interest (regarded as bait) and this combination is expressed in organisms or cultured cells. The BirA* releases biotinoyl-AMP into its immediate environment and the released compound labels lysine residues between the range of 5–10 nm to the bait protein. The biotinylation permits the use of harsh lysis conditions to enhance the solubility of proteins from intracellular compartments that are normally poorly soluble, such as the nuclear lamina, membranes or the chromatin. The biotinylated proteins are then trapped with streptavidin affinity, followed by mass spectrometry identification. Because the diameter of an average globular protein is 5–10 nm, the radius of labeling for the BioID technique benefits the biotinylation of proteins that are localized to the immediate intracellular environment of the bait and its direct binding partners [4].

For the characterization of proteome organization in living cells, Go et al. [1] employed the BioID approach towards the profiling of 234 baits for 32 different compartments of the cell. A total of 192 of the 234 baits passed quality control, leading to the establishment of 35,902 interactions among which 4424 unique proximity interactors were generated with high confidence. As for the localization of preys to specific intracellular compartments, the authors explored the assumption that prey proteins that share similar bait interaction might be localized to the same subcellular compartment, multiprotein complex or organelle. With the application of the NMF (non-negative matrix factorization) and SAFE (spatial analysis of functional enrichment) analytical pipelines, 3252 and 4145 of the total number of prey proteins were respectively localized to a minimum of 20 subcellular compartments. Each of the identified compartments by the SAFE and NMF also exhibited enrichment for specific protein motifs and domains. An example is the significant enrichment of the cell junction compartment of the cell for FERM and PDZ domains.

In an attempt to enhance their dataset exploration, the authors have designed the human cell map (https://cell-map.org/) which permits data viewing and searching regarding all profiled organelles, baits and prey proteins. Major features of the database includes its usage for the identification of queried bait-specific preys, the localization of a query bait to particular compartments of the cell based on similarity with the interactomes of other baits, and the identification of previously queried baits that have similar interactomes. Included among these features also is the ability to make comparison between the human cell map database and user BioID data. Although the BioID-predicted proteome compartmentalization appears to share high similarity with predictions made by fractionation and large-scale microscopy studies, the inability of the method to factor in the spatiotemporal dynamics of the human proteome in its predictions remains a limitation. To illustrate this, we compared the single-cell proteogenomic-based spatiotemporal prediction of the APPL1 localization from the human protein atlas (https://www.proteinatlas.org/) (Fig. 1) as reported by Mahdessian et al. [5], with the BioID-based predicted subcellular localization of the human cell map. Our observation showed that the scRNA-seq-based predictions that were obtained from the human protein atlas (mitotic spindle and the centrosome) were not included in the NMF- and SAFE-identified compartments of the APPL1. This suggests that upon specific cell signaling (yet to be delineated), there is a translocation of the protein from its initial subcellular compartments to mitotic structures, invariably making it a cell cycle-dependent protein [6].

**Fig. 1:**
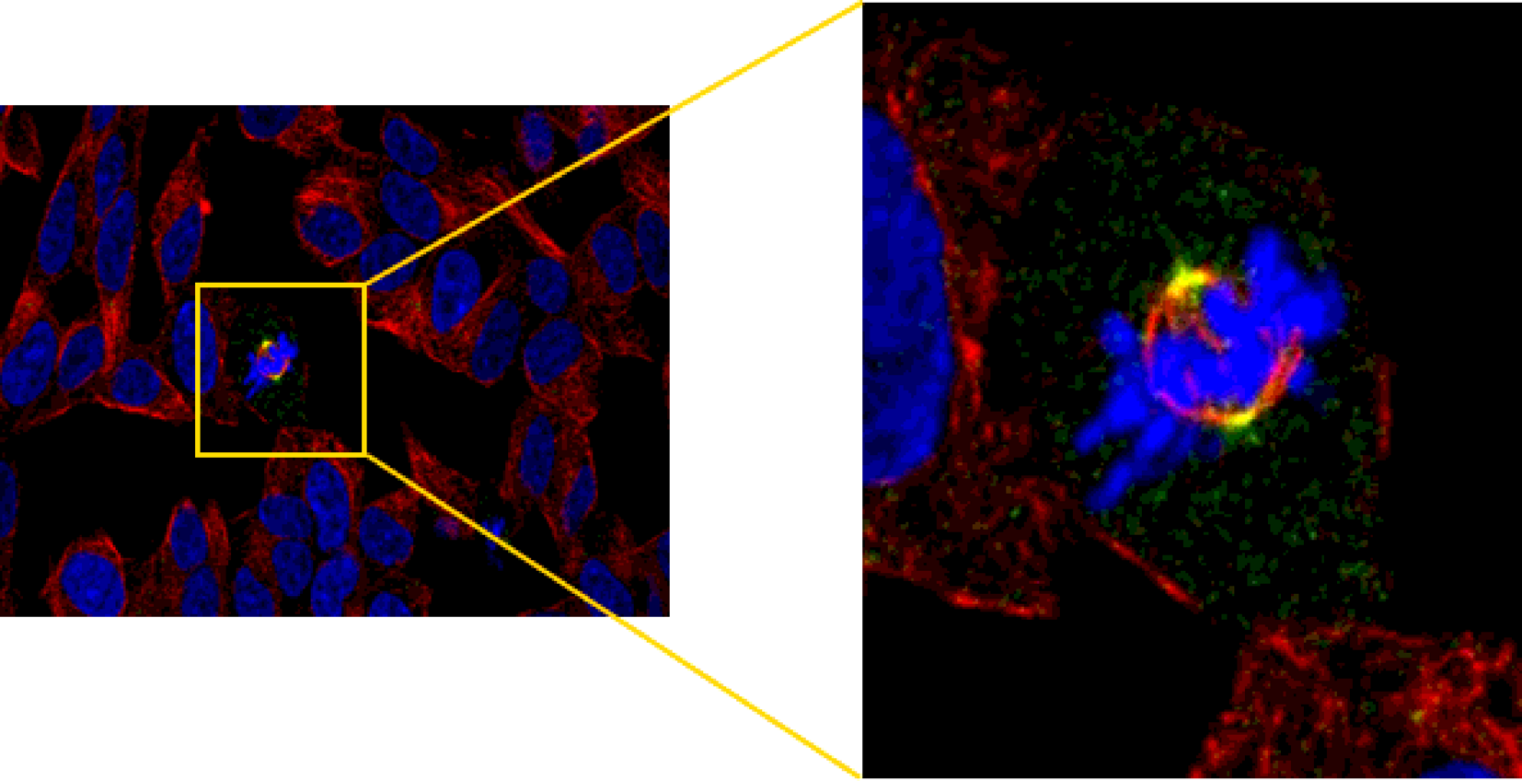
Immunofluorescence microscopy-resolved spatial distribution of the APPL1 in SH-SY5Y cell line, showing the mitotic spindle and centrosome localization of the protein. Localization to these mitotic structures suggest association with the cell cycle. (adapted from the human protein atlas)

In conclusion, the mass spectrometry in conjunction with biochemical fractionation and microscopy has been popularly applied in defining the proteomes of different cellular organelles, but most intracellular compartments of the cell have remained obstinate to such methods. Although, the specificity of the described BioID-based proximity labeling prediction for proteome compartmentalization exceeded those from previously reported approaches, limitations as a result of the spatiotemporal dynamics of the human proteome remains a factor to be considered in order to increase the specificity of predictions.

## Data Availability

Not applicable.
